# Alveolar Macrophage Dysfunction and Increased PD-1 Expression During Chronic SIV Infection of Rhesus Macaques

**DOI:** 10.3389/fimmu.2019.01537

**Published:** 2019-07-03

**Authors:** Ruth Hunegnaw, Zuena Mushtaq, Gospel Enyindah-Asonye, Tanya Hoang, Marjorie Robert-Guroff

**Affiliations:** Immune Biology of Retroviral Infection Section, Vaccine Branch, National Cancer Institute, National Institutes of Health, Bethesda, MD, United States

**Keywords:** alveolar macrophage, SIV, rhesus macaque, antibody-dependent phagocytosis, PD-1, FcγRIII, bronchoalveolar lavage

## Abstract

HIV infected individuals have been shown to be pre-disposed to pulmonary infections even while receiving anti-retroviral therapy. Alveolar macrophages (AMs) play a critical role in lung innate immunity, but contradictory results have been reported regarding their functionality following HIV infection. Here, using the SIV rhesus macaque model, we document the effect of SIV infection on the phenotypic and functional properties of AMs. Following infection with SIV_mac251_, AMs in bronchoalveolar lavage (BAL) sampled over 2- to 20-weeks post-infection (wpi) were compared to those in BAL samples from naïve macaques. AM expression of proinflammatory cytokines TNF-α, IL-6, IL-1β, and chemokine RANTES drastically increased 2-wpi compared to AMs of naïve macaques (*p* < 0.0001 for all), but dropped significantly with progression to chronic infection. Phagocytic activity of AMs 2-and 4-wpi was elevated compared to AMs of naive animals (*p* = 0.0005, *p* = 0.0004, respectively) but significantly decreased by 12-wpi (*p* = 0.0022, *p* = 0.0019, respectively). By 20-wpi the ability of AMs from chronically infected animals to perform SIV-specific antibody-dependent phagocytosis (ADP) was also diminished (*p* = 0.028). Acute SIV infection was associated with increased FcγRIII expression which subsequently declined with disease progression. Frequency of FcγRIII^+^ AMs showed a strong trend toward correlation with SIV-specific ADP, and at 2-wpi FcγRIII expression negatively correlated with viral load (*r* = −0.6819; *p* = 0.0013), suggesting a contribution to viremia control. Importantly, PD-1 was found to be expressed on AMs and showed a strong trend toward correlation with plasma viral load (*r* = 0.8266; *p* = 0.058), indicating that similar to over-expression on T-cells, PD-1 expression on AMs may also be associated with disease progression. Further, AMs predominantly expressed PD-L2, which remained consistent over the course of infection. PD-1 blockade enhanced SIV-specific ADP by AMs from chronic infection indicating that the PD-1/PD-L2 pathway may modulate functional activity of AMs at that stage. These findings provide new insight into the dynamics of SIV infection leading to AM dysfunction and alteration of pulmonary innate immunity. Our results suggest new pathways to exploit in developing therapies targeting pulmonary disease susceptibility in HIV-infected individuals.

## Introduction

Macrophages are found in tissues of infected individuals and play a continuous role in pathogenesis. Although earlier studies reported that macrophages were the major cell type initially infected by HIV (1), more recent studies have shown that CD4+ T cells are preferentially targeted by HIV/SIV at the site of transmission (2, 3). However, macrophages still serve as targets for the virus *in vivo* (4). They can sustain viral replication, disseminate virus, and serve as a viral reservoir post-infection (5–7). Cells of the macrophage/monocyte lineage vary greatly in phenotype, longevity, and in phagocytic, immunoregulatory, and secretory properties (8–11). Macrophages are categorized as classically (M1) or alternatively (M2) activated based on surface markers and functional role (12, 13). M1 macrophages mediate inflammatory responses against pathogens while M2 macrophages have anti-inflammatory properties, promoting tissue repair and remodeling (14). Alveolar macrophages (AM) in the lung uniquely express both M1 and M2 phenotypic markers, indicating ability to quickly respond to pathogens but also prevent immune activation in response to harmless antigens that enter the alveolar lumen (15).

AM express CD4 and chemokine receptors making them vulnerable to HIV infection (16). However, macrophages may be poorly susceptible to HIV induced cytopathic effects. Minimal consequences of HIV infection on the macrophage transcriptome were observed (17); in contrast expression of HIV Nef and gp120 envelope induced macrophage activation (18, 19). Indirect activation can also occur by exposure of uninfected macrophages to viral gene products or cytokines from other infected cells (20).

AM are important lung phagocytes (21), yet AM obtained from HIV-infected individuals have shown contradictory results regarding the impact of viral infection on phagocytosis. Some studies have shown impaired phagocytosis of opportunistic pathogens by AM (22–26); others have reported no change in AM phagocytic activity during infection (27, 28). Such variations in outcome may result from differences in length of infection or reliance on the use of monocyte derived macrophages (MDM) and infected cell lines which may not ideally represent clinical situations. Macrophages can utilize Fcγ receptors to internalize antibody-opsonized virions or infected cells, potentially leading to antibody-mediated clearance of infectious material (29). Antibody-dependent phagocytosis (ADP) by AM contributes to protection against viral infections such as influenza (30), West Nile (31), adenovirus (32), and SARS coronavirus (33). However, ADP-mediated protection by macrophages against HIV infection has not been observed.

Here we investigated the dynamics of SIV-related changes in AM activity and function by sampling bronchoalveolar lavage (BAL) from SIV infected rhesus macaques during acute and chronic infection. The AM response to SIV infection consisted of phenotypic changes and alterations in proinflammatory responses, ability to respond to gp120 antigen, and phagocytic activity. FcγRIIIb expression on AM was linked to SIV-specific ADP and viral control during acute infection. Novel results showed increased expression of Programmed Cell-Death-1 (PD-1) on AM from chronically infected macaques and positive correlations between PD-1 expressing AMs and SIV viremia. We believe this is the first report of PD-1 expression on AMs of SIV infected macaques. Our results suggest associations between PD-1 expression, macrophage dysfunction, and lack of viremia control.

## Materials and Methods

### Animals and Challenge

Forty-nine female Indian rhesus macaques used in this study were housed in the NCI Animal Facility, Bethesda, MD, under protocol VB-020. The NCI Facility is accredited by the Association for Assessment and Accreditation of Laboratory Animal Care International, and its Animal Care and Use Committee approved all animal experiments prior to study initiation. Standard practices followed recommendations made in the Guide for the Care and Use of Laboratory Animals of the United States, National Institutes of Health, and the Weatherall report. Thirty macaques were used as naïve controls and 19 macaques were challenged weekly intravaginally using a repeated low-dose of SIV_mac251_ (800 TCID_50_). Infection was confirmed for 14 of the macaques with plasma viral loads of ≥50 SIV RNA copies/ml as assessed by droplet digital PCR (Chung et al., manuscript in preparation). The remaining five macaques were infected intrarectally with a single high dose of SIV_mac251_ (4000 TCID_50_). Out of the 19 infected macaques, 9 were euthanized after 2 wpi and the remaining 10 infected macaques were monitored longitudinally for up to 20 wpi.

### BAL Collection and Processing

BAL samples were obtained using standard techniques (34). Briefly, rhesus macaques were anesthetized, and an endotracheal tube was inserted through which sterile saline solution (10 ml/kg) was instilled. Suction was applied to recover the instilled fluid and the lung lavage was collected in sterile conical tubes. Cells were pelleted by centrifuging at 1600 RPM for 10 min at 4°C and washed in 25 mL cold PBS. Centrifugation was repeated, and cells were resuspended for counting. Cells showed ~90% viability as determined by trypan blue staining.

### AM Enrichment and Stimulation

BAL cells were enriched for AMs by depleting EPCAM^+^, CD2^+^, and CD20^+^ cells. The following reagents were used: rabbit polyclonal anti-EPCAM (N3C3) (GeneTex, Irvine, CA); CD2 and CD20 MicroBeads for non-human primate (Miltenyi Biotec, Auburn, CA). Depletion was performed using MACS cell separation technology according to the manufacturer's instructions (Miltenyi Biotec, Auburn, CA). Enriched AMs were stimulated for 6 h in 500 μl R-10 media (RPMI + 10% FBS + 5% Pen-Strep + 5% Anti-Anti) containing purified native HIV-1 envelope gp120 (Advanced Bioscience Laboratories, Inc., Rockville, MD) at a concentration of 200 nM or LPS (ThermoFisher Scientific, Waltham, MA) at 500 ng/ml concentration. For experiments assessing cytokine production by PD-1^+^ AMs, cells were stimulated with 10 μg/ml LPS in the presence of Golgistop (1 μl, BD Biosciences) for 18 h prior to intracellular staining.

### AM Antibody Dependent Phagocytosis (ADP) Assay

ADP activity was measured as previously described (35). SIV_mac251_ gp120 was biotinylated with a Biotin-XX microscale protein labeling kit (Life Technologies, Grand Island, NY) and incubated with a 10-fold dilution of 1 μg Avidin coated Sky Blue fluorescent beads (0.8 μm diameter; Spherotech, Lake Forest, IL) overnight at 4°C. Enriched AMs were plated in a U-bottom 96 well plate at 40,000 cells/well and undiluted BAL-F was added. To determine phagocytic activity of AM independent of SIV specific antibody, BAL-F from naïve macaques was used. For SIV-specific ADP, autologous BAL-F from infected macaques was used. The bead-gp120 mixture was brought to a final 50-fold dilution in R-10 media and 50 μl was added to the cells and incubated for 3 h at 37°C. For PD-1 blocking experiments, BAL samples collected from macaques at 20 wpi were enriched for AMs as described above and incubated with either 10 μg/ml Ultraleaf purified anti-human PD-1 antibody (EH12.2H7, BioLegend) or 10 μg/ml mIgG1 isotype control (Sigma-Aldrich). Cells incubated with naïve BAL-F were used for normalization. In all cases, after 3 h of incubation, 70 μl of 2% paraformaldehyde was added for fixation. Fluorescent bead uptake was assessed using a BD Biosciences LSRII flow cytometer. Bead uptake specifically by AM was made possible by focusing on cells that were autofluorescent in the FITC channel. The phagocytic score of each sample was calculated by multiplying the frequency of bead-positive cells by the degree of phagocytosis measured as mean fluorescence intensity (MFI) and dividing by 10^4^. Values were normalized to background values (cells and beads with either PBS or naïve BAL-F) by dividing the phagocytic score of the test sample by the phagocytic score of the background samples.

### AM Staining

Mouse anti-non-human primate or anti-human fluorochrome-conjugated monoclonal Abs (except where specified otherwise) known to cross-react with Rhesus macaque antigens were used in this study. The following antibodies were used for surface staining: BV711 anti-CD4 (L200), BV786 anti-CD45 (D058-1283), BUV395 anti-CD3 (SP34-2), BUV496 anti-CD16 (3G8), BV711 anti-CD32 (FLI8.26/8.26), BUV737 anti-CD64 (10.1), BUV805 anti-CD14 (M5E2), BV605 anti-CD80 (L307), PE anti-CD86 (B7.2) (from BD Biosciences, San Jose, CA); PE-Cy7 anti-CD206 (19.2), APC-Cy7 anti-CD11b (ICRF44), Blue Dead cell stain kit for cell viability (from ThermoFisher Scientific, Waltham, MA); AF700 anti-CD11b (ICRF44), BV650 anti-CD11c (3.9), APC-Fire anti-PD-1 (EH12.2H7), BV786 anti PD-L1 (29E.2A3), APC anti-PD-L2 (24F.10C12), PE-Cy5 anti-HLA-DR (L243), BV510 anti-CD163 (GH1/61) (from BioLegend, San Diego, CA). For intracellular staining, BB515 anti-RANTES (2D5), APC anti-MIP-1α (11A3), BV421 anti-MIP-1β (D21-1351) (BD Biosciences, San Jose, CA); AF700 anti-IL-6 (MQ2-13A5) (ThermoFisher Scientific, Waltham, MA); rat monoclonal Ax488 anti-IL-10 (JES3-97D), BV605 anti-TNF-α (Mab11) (BioLegend, San Diego, CA) were used. The Blue LIVE/DEAD™ viability dye (ThermoFisher Scientific, Waltham, MA) was used to exclude dead cells. For staining, surface antibodies were added to cells and incubated for 25 min at room temperature (RT). Cells were then washed with PBS prior to resuspending in BD Cytofix/Cytoperm™ and incubating for 15 min at RT. Cells were then washed with BD Permwash, and incubated with intracellular staining antibodies for 25 min at RT. Finally, cells were washed with PBS and staining data were acquired using the 5 laser BD FACSymphony (BD Biosciences, San Jose, CA). Fluorescence Minus One (FMO) and isotype controls were used to confirm the phenotype and cytokine expressing population of AMs (Figure S1). Data analyses were performed with FlowJo (version 10.5, TreeStar, Inc Ashland, OR) software.

### Quantification of IgGs by ELISA

Antibodies in BAL-F were measured as follows: wells of Greiner high-binding $\frac{1}{2}$ area 96-well plates were coated overnight at 4°C with 100 ng/well of SIV_mac251_ gp120 (for determining gp120-specific IgG) or 50 ng/well of anti-rhesus IgG (Alpha Diagnostics) (for determining total IgG) in sodium bicarbonate buffer (pH 9.6) (Sigma-Aldrich, St. Louis, MO). Wells were blocked with 200 μl of 1% BSA diluent/blocking solution (KPL) in distilled water for 2 h at RT. BAL-F (50 μl) was added and incubated for 1 h at 37°C. Env-specific IgG derived from purified serum IgG obtained from SIV_mac251_-infected macaques and quantified as previously described (36) was used to generate a standard curve for Env-specific IgG. Purified rhesus IgG (Non-Human Primate Reagent Resource) was used to generate a standard curve for total IgG. Plates were washed 5 times with 1X wash solution (KPL). Horseradish peroxidase-labeled goat anti-monkey IgG (50 μl at a 1:10,000 dilution; Alpha Diagnostics) was added, and plates were incubated for 1 h at 37°C. After washing as described above, 50 μl of 3,3′,5,5′-tetramethylbenzidine (TMB) peroxidase substrate (KPL) was added for 10–20 min at RT. Color development was stopped with 50 μl 1 M Phosphoric acid (Sigma), and plates were read at 450 nm by using a Biotek plate reader. SIV gp120-specific antibody levels were expressed as ng gp120-specific IgG/μg total IgG.

### Statistical Analysis

Statistical analysis was performed using one-way ANOVA or 2way ANOVA with the Tukey multiple comparisons test as indicated in the Figure legends. The Wilcoxon matched-pairs signed rank test was also used where indicated. Correlation analyses were performed by non-parametric Spearman correlations. Statistics were generated using GraphPad Prism.

## Results

### Identification and Phenotyping of AMs Over the Course of SIV Infection

**Figure 1:**
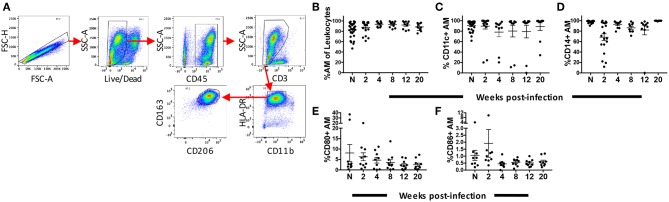
Identification and phenotyping of AMs over the course of SIV infection. BAL cells were collected from either naïve (*n* = 30) rhesus macaques or SIV infected macaques at weeks 2 (*n* = 19), 4 (*n* = 10), 8 (*n* = 10), 12 (*n* = 10), and 20 (*n* = 10). Recovered cells were stained and analyzed by flow cytometry. **(A)** Gating strategy used to identify AMs. Doublets and dead cells were excluded, and leukocytes were discriminated from epithelial cells by CD45 expression. Gates were extended to include highly granular and autofluorescent AMs. CD3 expression excluded lymphocytes. AMs were identified as FSC^high^SSC^high^, CD11b^int^HLA-DR^high^CD163^+^CD206^+^ **(B)** Frequency of CD163^+^CD206^+^ AMs in total BAL leukocytes. **(C–F)** Percentage of AMs expressing CD11c, CD14, CD80, and CD86, respectively. *N* = naïve.

Rhesus macaques were infected with SIV_mac251_ and BAL was collected at 2, 4, 8, 12, and 20-wpi. Nineteen macaques were sampled at 2-wpi and 10 for the subsequent time points. BAL samples were also obtained from 30 naïve macaques. AMs from BAL were characterized by flow cytometry as HLA-DR^hi^, CD11b^int^, and CD163^+^CD206^+^ (37) (Figure 1A). The mean frequency of AMs was >80% of leukocytes found in the lavage samples, consistent with other reports on BAL specimens from naive rhesus macaques (37). No significant change in the frequency of AMs was observed over the 20 weeks of infection (Figure 1B). Expression of CD11c and CD14 has been shown to identify AM phenotypes with changes in expression occurring during the fibrotic stage of lung disease (38) Here, no significant changes in AM expression of CD11c or CD14 over the time period studied were observed (Figures 1C,D). We also did not observe changes in the percentage of AMs expressing the costimulatory molecules, CD80 and CD86, indicating that any inflammatory responses recorded may not be associated with the expansion of these cell subsets (Figures 1E,F).

### Proinflammatory Cytokine and Chemokine Expression by AM Peaks at the Acute Phase of SIV Infection

**Figure 2:**
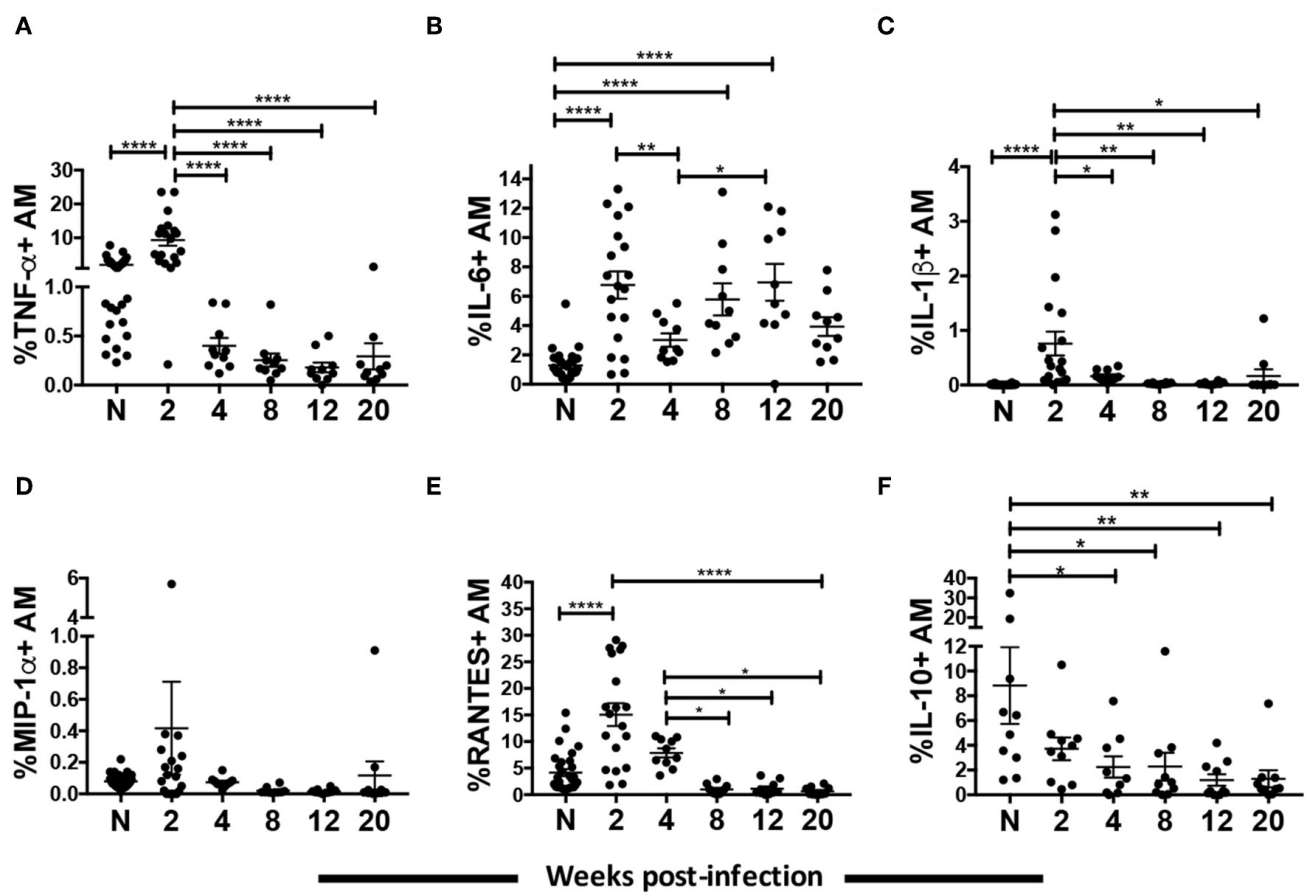
AM cytokine/chemokine expression over the course of SIV infection. BAL cells collected from naïve (**A–E**, *n* = 30; **F**, *n* = 10) or SIV infected rhesus macaques at 2 (**A–E**, *n* = 19; **F**, *n* = 10), 4, 8, 12, and 20 (*n* = 10) weeks post-infection were stained for intracellular expression of cytokines/chemokines. **(A–F)** Percentage of AMs expressing TNF-α, IL-6, IL-1β, MIP-1α, RANTES, and IL-10, respectively. Data are represented as mean ± SEM. Statistical differences were determined using one-way ANOVA and Tukey's multiple comparisons test. (^*^p < 0.05, ^**^p < 0.01, ^****^p < 0.0001). N, naïve.

Macrophages secrete proinflammatory cytokines and chemokines in response to HIV infection (39, 40). However, a clear dynamic of the cytokine and chemokine response by macrophages over the course of HIV/SIV infection has not been shown. Here we characterized cytokine levels in AM collected from SIV infected rhesus macaques between 2 and 20-wpi and compared them to levels in AMs from naïve macaques. A strong proinflammatory response was seen during the acute phase of infection. Expression of TNF-α, IL-6, and IL-1β in AMs was significantly increased by 2-wpi (*p* < 0.0001 for all; Figures 2A–C). By 4-wpi expression levels were significantly lower. Cytokine expression remained low until the 20-wpi time point except for IL-6 expression, which increased significantly by 12-wpi compared to 4-wpi (Figure 2B). The IL-6 expression level at 20-wpi was not significantly higher than levels seen in naïve populations. Similarly, a higher level of the chemokine RANTES was found in AMs from macaques at 2-wpi (Figure 2E). Expression decreased to levels comparable to naïve samples by 4-wpi and remained low until the 20-wpi time point (Figure 2E). MIP-1α expression did not change over the course of infection (Figure 2D). Overall, we observed a transient increase in the proinflammatory cytokine responses, which decreased to levels comparable to those of naïve macaques during the chronic phase of infection. To assess whether AMs were leaning toward an anti-inflammatory role during chronic infection, we evaluated the intracellular expression of IL-10 by AMs (Figure 2F). AMs play an important role in maintaining an anti-inflammatory environment in the lung (21, 41). Indeed, IL-10 expressing AMs were detected in naïve animals and the frequency of IL-10+ cells was significantly higher compared to that of acutely infected animals (Figure 2F). Interestingly, the percentage of IL-10 expressing AMs further decreased significantly as infection progressed into the chronic stage (Figure 2F). Thus, SIV infection was associated with a significant spike in the AM proinflammatory response by 2-wpi, which waned in chronic infection. In addition, the low proinflammatory cytokine response in chronic infection was not associated with an increase in IL-10-expressing AMs.

### Chronic SIV Infection Is Associated With Diminished Response of AMs to gp120 and LPS Stimulation

**Figure 3:**
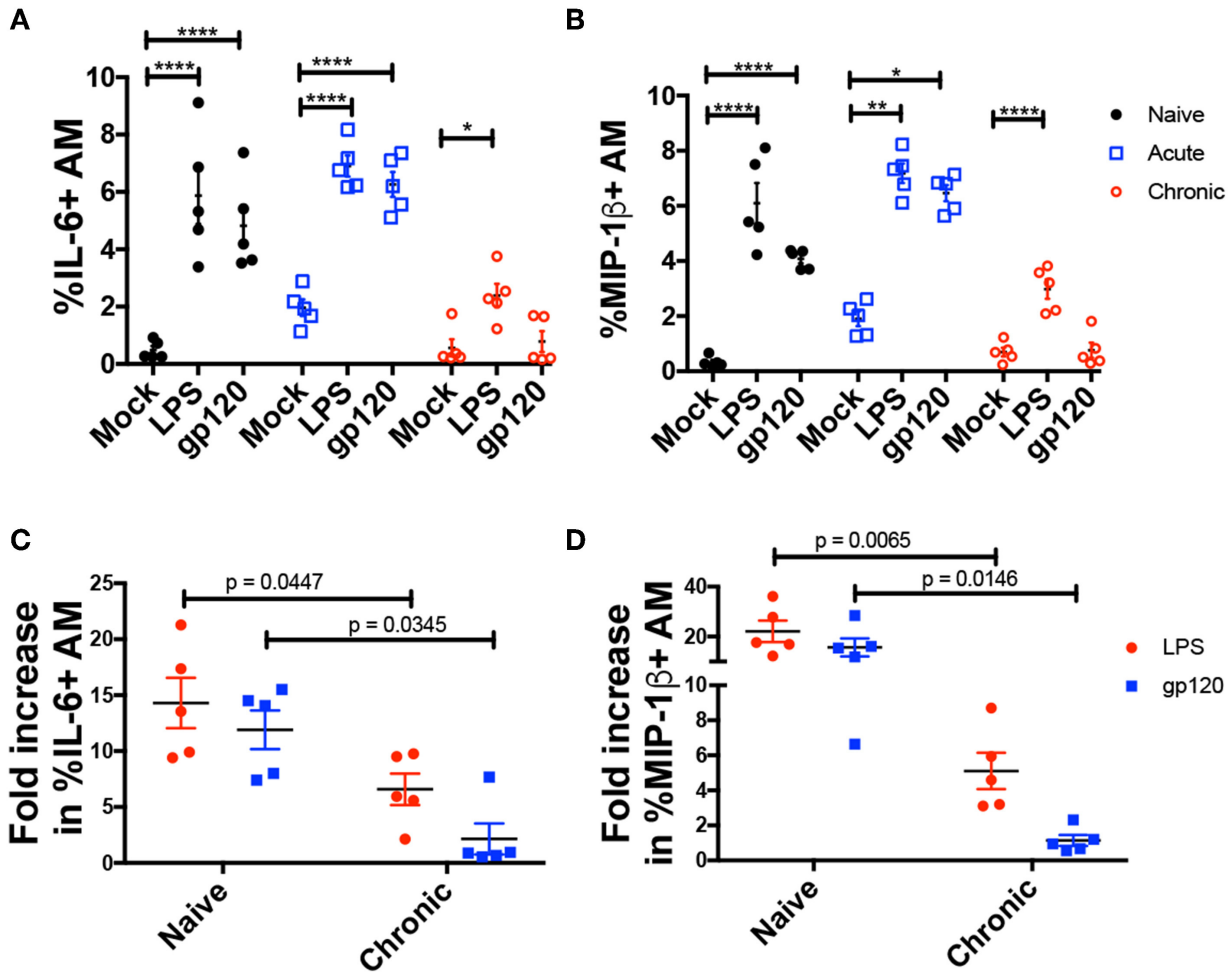
AMs from chronically infected macaques show diminished response to LPS and gp120 stimulation. BAL cells from 5 macaques at naïve or acute and chronic infection stages were cultured with LPS or native SIVmac251 gp120 protein for 6 h. Cells were then stained and analyzed by flow cytometry for cytokine/chemokine expression. **(A,B)** Percent of AMs expressing IL-6 and MIP-1β, respectively. **(C,D)** Fold increase in frequency of **(C)** IL-6^+^ and **(D)** MIP-1β^+^ AMs. Data are represented as mean ± SEM. Statistical differences were determined using 2way ANOVA and Tukey's multiple comparisons test. (^*^p < 0.05, ^**^p < 0.01, ^****^p < 0.0001). N, naïve.

To investigate AM activation, BAL cells obtained from naïve and acute and chronically infected macaques at weeks 2, 4, 8, 12, and 20 wpi, were incubated with native gp120 from R5 tropic SIV or LPS for 6 h, and intracellular expression of MIP-1β and IL-6 was assessed (Figures 3A,B). Both gp120 and LPS induced comparable levels of MIP-1β and IL-6 in AMs from naive and acutely-infected animals (Figures 3A,B). However, in chronically infected macaques gp120 did not induce intracellular IL-6 or MIP-1β expression in AMs (Figures 3A,B), and LPS did not stimulate expression of either factor to the level induced in naïve animals suggesting a dysfunctional state of the AMs (Figures 3C,D).

### Changes in the Phagocytic Function of AM Over the Course of SIV Infection

**Figure 4:**
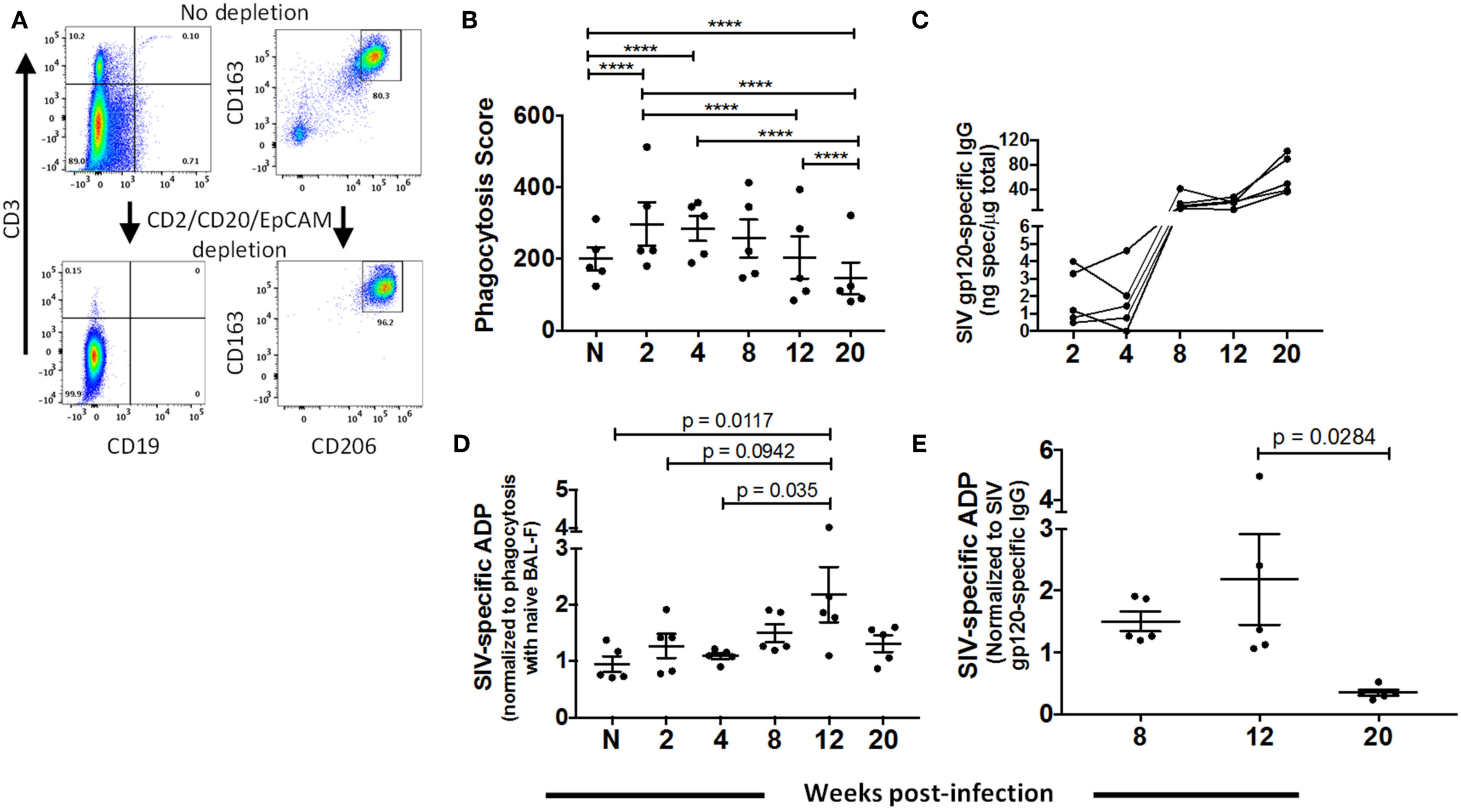
Dynamics of AM phagocytic activity over the course of SIV infection. BAL cells were collected from naïve (*n* = 5) or SIV-infected (*n* = 5) rhesus macaques at 2, 4, 8, 12, and 20 weeks PI for assay of phagocytic activity and ADP as described in Methods. **(A)** Representative gating showing depletion of lymphocytes (left panel) and percentage of AMs (right panel) from enriched (CD2^+^CD20^+^EPCAM^+^ depleted) and non-enriched live BAL cells. **(B)** Phagocytosis by AMs over the course of SIV infection. **(C)** SIV_mac251_ gp120 specific IgG in BAL-F over the course of infection for each sample. **(D)** AM SIV-specific ADP in the presence of autologous BAL-F normalized against the phagocytic activity in the presence of naïve BAL-F. **(E)** SIV-specific ADP score normalized against SIV gp120 specific IgG at the indicated time points. Data in **(B)**, **(D)**, and **(E)** are represented as mean ± SEM. Statistical differences were determined using 2 way ANOVA and Tukey's multiple comparisons test (^****^p < 0.0001).

To assess whether AMs contribute to clearance of SIV, we evaluated their phagocytic activity and ability to perform ADP over the course of SIV infection using BAL samples from 5 naïve animals and 5 SIV-infected macaques sampled between 2 and 20-wpi. While the BAL samples contained a high percentage of AMs (Figure 1B), the cells were further enriched for AMs using MACS by depleting CD2, CD20, and EPCAM positive cells (Figure 4A). To assess phagocytic capacity independent of SIV antibody, enriched cells were incubated with SIV_mac251_ gp120-coated fluorescent beads and BAL-F from naïve macaques prior to quantification of bead uptake. A significant increase in phagocytic activity was detected during the acute phase, 2 and 4-wpi (Figure 4B). At 8-wpi, phagocytosis decreased and was comparable to naïve cells. Activity further decreased significantly at the 12 and 20-wpi time points compared to the acute and naïve time points (Figure 4B).

Prior to evaluating ADP by AMs, we assessed SIV gp120-specific IgG in BAL-F (Figure 4C). Specific IgG was detected at 8-wpi with levels maintained or continuing to rise until 20-wpi. To determine SIV-specific ADP, AMs from naïve macaques or sampled over the course of SIV infection were incubated with antigen-coated beads along with autologous BAL-F. Phagocytosis scores were normalized against the score obtained using naïve BAL-F to highlight phagocytic activity associated with the presence of SIV-specific antibody (Figure 4D). Significantly higher phagocytic scores were detected at 12-wpi compared to those at naïve and 4-wpi time points, indicating that AMs could perform SIV-specific ADP (Figure 4D). To further assess ADP by AMs in chronic infection, phagocytic scores at chronic time points (Figure 4D) were normalized against SIV-specific antibody levels (Figure 4C). Results showed that in the presence of comparable antibody levels, AMs mediated ADP activity at 12-wpi. However, this activity was significantly compromised by 20 week PI (Figure 4E). Thus, AM are capable of gp120-specific ADP for a limited time period during chronic infection. This diminished functional capacity of AMs in the chronic stage further highlights the dysfunction observed upon LPS and gp120 stimulation (Figure 3).

### Dynamics of FcγR Expression on AMs Over the Course of SIV Infection and Association With ADP

**Figure 5:**
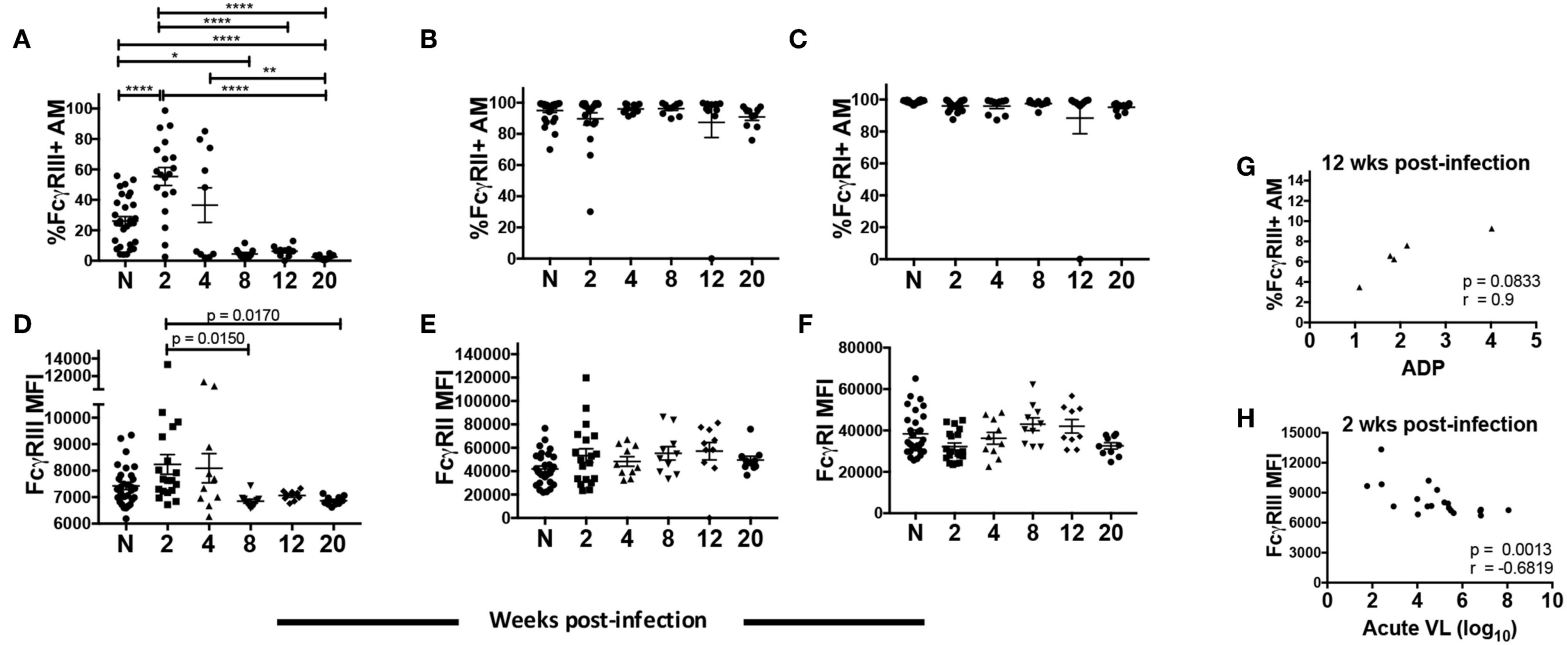
AM FcγR expression and role in SIV-specific ADP and viral load control. BAL cells collected from naïve (*n* = 30) or SIV infected rhesus macaques at 2 (*n* = 19), 4, 8, 12, and 20 (*n* = 10) weeks post-infection were stained. **(A–C)** Percentage of AMs expressing FcγRIII, FcγRII, and FcγRI, respectively. **(D–F)** MFI of FcγRIII, FcγRII, and FcγRI expression in AMs. **(G)** Phagocytic activity of AMs was measured in 5 infected macaques at 2, 4, 8, 12, and 20 wpi. Correlation of the frequency of FcγRIII^+^ AMs with SIV-specific ADP at 12-wpi (*n* = 5). **(H)** Surface expression of FcγRIII on AMs was assessed from a total of 19 acutely infected macaques. Correlation of AM FcγRIII expression at 2-wpi with acute viral load (*n* = 19). Data in **(A–F)** are represented as mean ± SEM. Statistical differences were determined using one-way ANOVA and Tukey's multiple comparisons test. **(A–F)** (^*^p < 0.05, ^**^p < 0.01, ^****^p < 0.0001). Correlation statistics were generated using Spearman correlation. N, naïve.

Three classes of FcγR are constitutively expressed on AMs with an active role in protecting against pathogens in the airway: high-affinity FcγRI (CD64) and low-affinity FcγRII (CD32) and FcγRIII (CD16) (42). FcγRIII occurs in two isoforms: FcγRIIIa (expressed mainly on NK cells and macrophages) and FcγRIIIb (expressed on neutrophils) (43–45). Infected MDMs have shown compromised expression of the γ signaling chain of FcγRIIIa, possibly leading to altered phagocytic activity (46). Monocytes from chronically infected individuals have also shown reduced expression of FcγRIIIa and diminished phagocytic activity (47). However, thus far no correlations have been found between ADP and HIV/SIV viral load, CD4 count or FcγR expression. Given the unique features of AMs compared to other tissue macrophages and monocytes, and the importance of phagocytic function in the lung, we investigated whether there was altered FcγR expression on AMs over the course of SIV infection and any association with ADP. BAL from naive macaques contained over 80% of FcγRI^+^ and FcγRII^+^ AMs (Figures 5B,C). Both the frequencies and MFI of FcγRII and FcγRI remained high and unchanged over the course of infection (Figures 5B,C,E,F). In contrast, the mean frequency of FcγRIII^+^ cells was 30% in naïve macaques. At 2-wpi there was a significant increase in FcγRIII^+^ AM frequency but levels significantly decreased by 8-wpi (Figure 5A). The low frequency of FcγRIII^+^ AMs was maintained until 20-wpi. A similar increase in AM FcγRIII MFI was not observed in the acute phase of infection compared to naïve animals (Figure 5D). However, expression levels were significantly reduced at the 8 and 20-wpi time points compared to levels at 2-wpi (Figure 5D) in concert with the decreased frequencies observed. The SIV-specific ADP observed at 12-wpi (Figure 4E) exhibited a strong trend toward positive correlation with the percentage of FcγRIII^+^ AMs at the same time point (Figure 5G). Further, the FcγRIII MFI at 2-wpi (Figure 5D) negatively correlated significantly with acute plasma viral load (Figure 5H). These data suggest that FcγRIII plays an important role in SIV-specific ADP and that its expression is associated with early viral load control.

**Figure 6:**
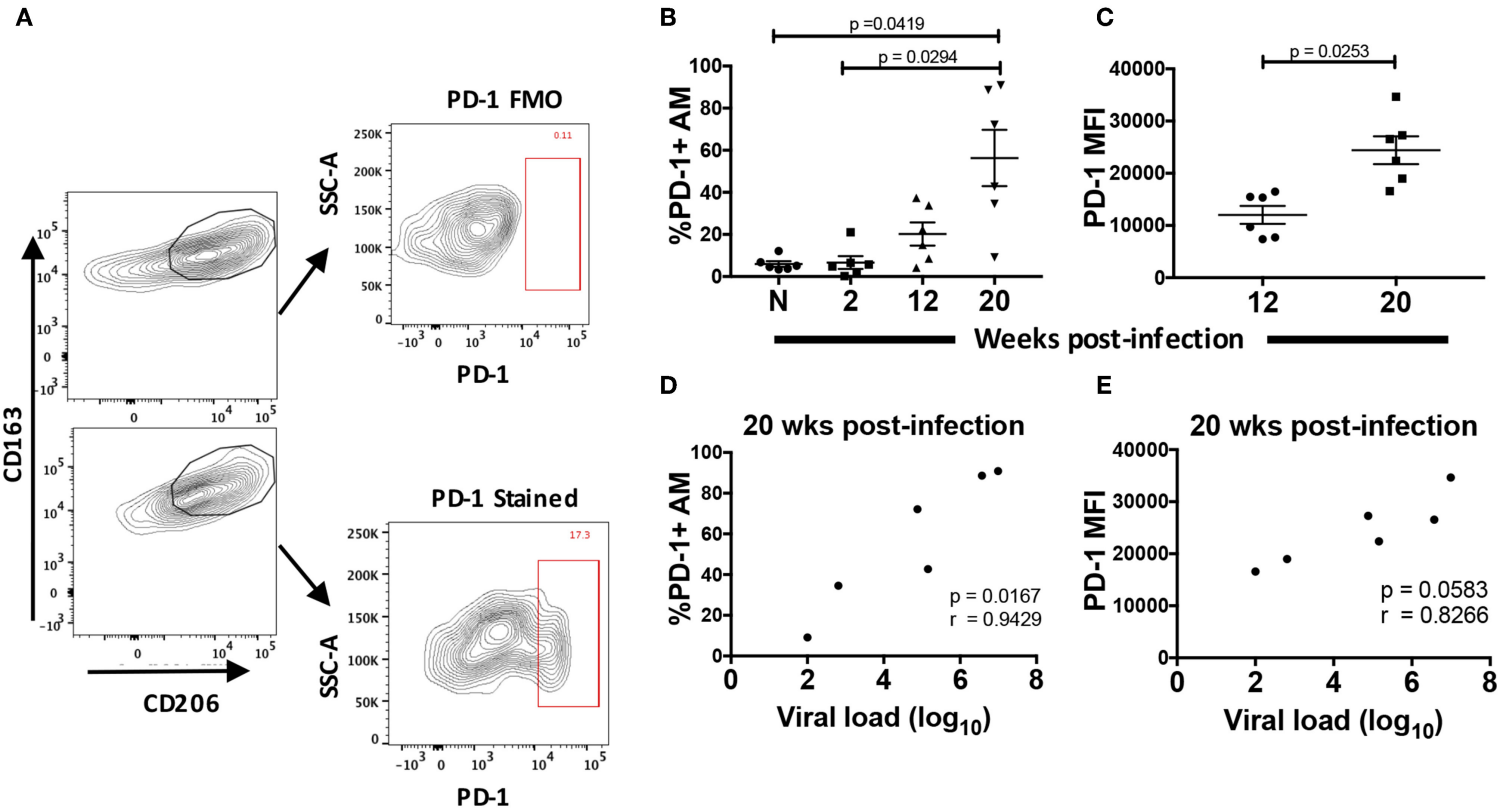
Surface expression of PD-1 is increased in chronically SIV-infected rhesus macaques. PD-1 expression was analyzed on AMs taken from 6 naïve macaques or at 2, 12, and 20 wpi. **(A)** Gating strategy showing a representative PD-1^+^ stained population. **(B)** Frequency of PD-1^+^ AMs over the course of SIV infection and **(C)** MFI of PD-1^+^ AMs at 12 and 20-wpi. **(D)** Correlation of percentage of PD-1^+^ AMs or **(E)** PD-1 MFI with viral load at 20-wpi. Data in **(B)** and **(C)** are represented as mean ± SEM. Statistical differences were determined using one-way ANOVA and Tukey's multiple comparisons test. Correlation statistics were generated using Spearman correlation. *n* = 6 macaques for all. **(B–E)** N, naïve.

To characterize the dysfunction observed in AMs from chronically infected macaques, we looked for parallels with T cell exhaustion. Since the initial description of T cell exhaustion in chronic viral infections (48, 49), common phenotypic and functional properties have been attributed to a dysfunctional state regardless of the type of pathogen. High and prolonged expression of inhibitory receptors are a key feature of T cell exhaustion in chronic viral infections (50). In particular, overexpression of the PD-1 receptor on CD8^+^ T-cells plays a major role in T cell dysfunction associated with HIV infection (51–53). Here, we identified PD-1^+^ AMs in rhesus macaque BAL and assessed changes in its expression on AMs from 6 macaques over the course of SIV infection (Figure 6A). When the frequency of PD-1^+^ AMs was assessed in naive macaques and compared to frequencies in SIV infected animals, five out of six SIV^+^ macaques showed an increase in frequency (Figure 6B). The PD-1^+^ AM frequency at 20-wpi was significantly different from both naïve and 2-wpi time points: *p* = 0.0419 and *p* = 0.0294, respectively. AMs at 20-wpi also displayed a significant increase in PD-1 MFI signifying increased expression of the receptor (Figure 6C). Furthermore, the percentage of PD-1^+^ AMs correlated positively with viral load at 20-wpi (Figure 6D). We also observed a strong trend toward correlation between PD-1 MFI and viral load at 20-wpi (Figure 6E), indicating an association between PD-1 expression in AMs and SIV disease progression.

### Blockade of PD-1 Improved Phagocytic Activity of Macrophages

**Figure 7:**
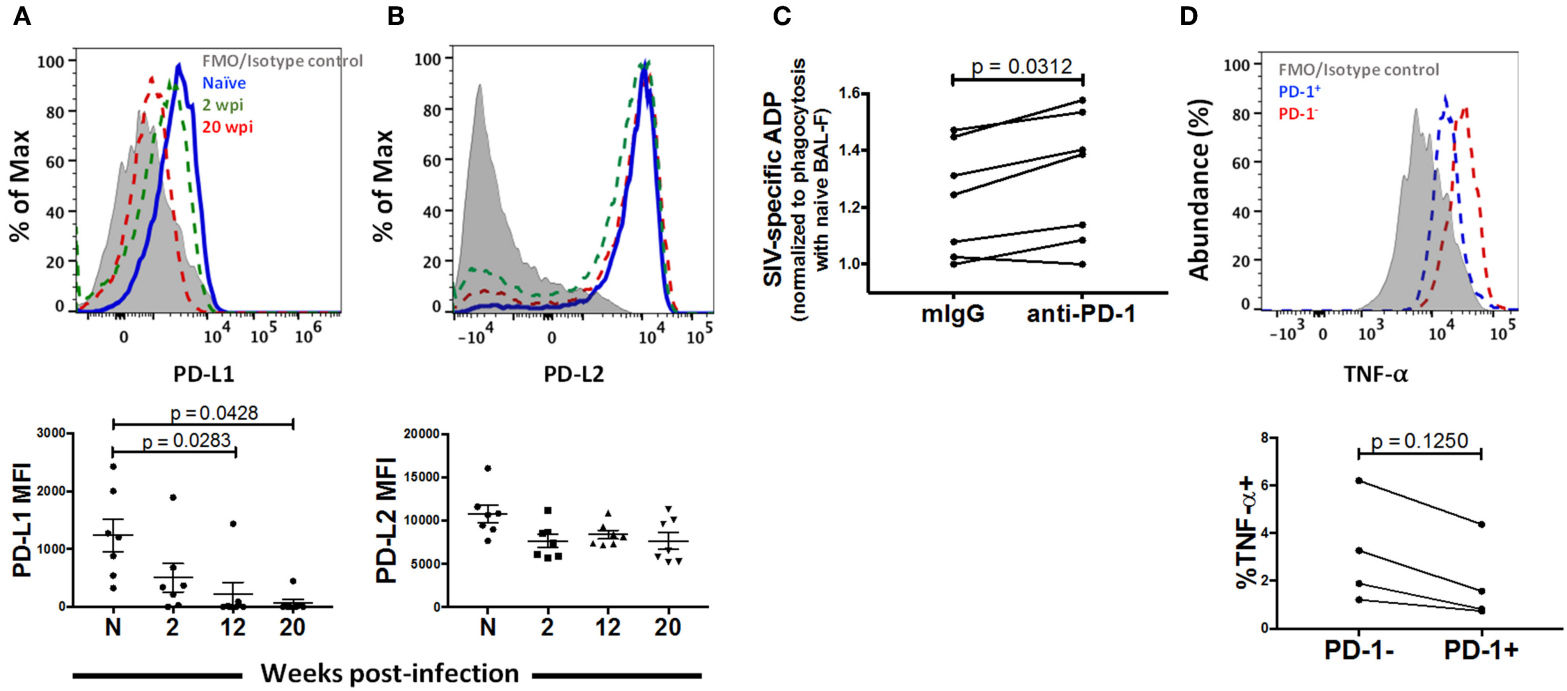
PD-1 blockade restores phagocytic activity in chronically infected macaques. Expression of **(A)** PD-L1 and **(B)** PD-L2 on AMs after 2, 12, and 20 wpi. *n* = 7. Paired one-way ANOVA with Tukey's multiple-comparisons correction. **(C)** Phagocytosis score of MACS sorted AMs treated with either mIgG or anti-PD-1 antibody. *n* = 7. Wilcoxon matched-pairs signed rank test. **(D)** AMs from macaques at 20 wpi were stimulated with LPS for 18 h and TNF-α expression was compared between the PD-1^+^ and PD-1^−^ populations of AMs. *n* = 4. Wilcoxon matched-pairs signed rank test. N, naive.

PD-1 has two well-known ligands via which inhibitory effects have been reported to occur: Programmed Death -Ligand 1 (PD-L1) and Programmed Death Ligand 2 (PD-L2). While PD-L1 has been shown to be expressed broadly in both hematopoeitic and non-hematopoeitic cells, a more restricted expression pattern has been documented for PD-L2, namely antigen presenting cells (APC) (54). In order to identify a potential role for the PD-1 pathway on AM function, we looked for expression of these ligands on AMs after SIV infection. AMs from naïve macaques predominantly expressed high levels of PD-L2 (Figure 7B). This expression decreased modestly, but not significantly, after SIV infection and remained consistent thereafter. On the other hand, we observed minimal expression (~10 fold lower than that of PD-L2) of PD-L1 on AMs from naïve macaques (Figure 7A). Further, PD-L1 expression significantly decreased over the course of SIV infection. These results indicate that AM dysfunction is more likely associated with increased PD-1 expression rather than changes in the expression of its ligands and may occur through the PD-1/PD-L2 pathway.

To identify whether PD-1 blockade can rescue AM phagocytosis, we examined the phagocytic activity of AMs from 7 chronically infected macaques in the presence of PD-1 blocking antibody or mouse IgG as control. Interestingly, we found higher phagocytic scores in the PD-1 blockade group in 6 out of the 7 animals (*p* = 0.0312), indicating that PD-1 expression is likely mechanistically linked to impairment of phagocytic activity (Figure 7C). We further assessed cytokine expression of PD-1 expressing AMs. We stimulated AMs from chronically infected macaques with 10 μg/ml LPS and compared cytokine expression between PD-1^+^ and PD-1^−^ populations. Although not significant, we found lower expression of TNF-α on PD-1 expressing cells compared to the PD-1^−^ population (Figure 7D). Comparisons with additional cytokines and chemokines (IL-6, IL-1β and RANTES) are not reported here as expression of these cytokines could not be detected in the AMs from the infected macaques that were tested. Taken together, these data strongly suggest that PD-1 expression on AMs during SIV infection may be a marker of dysfunction.

## Discussion

Our results show that in the SIV macaque model, the pro-inflammatory response of AMs to SIV infection peaks in the acute phase of infection and is diminished in the chronic phase as observed following stimulation with LPS or gp120. We report that AMs from SIV infected macaques can perform ADP early in infection, but this activity diminishes in the chronic phase. In novel findings, we report an elevated percentage of PD-1^+^ AMs in chronically infected macaques, which positively correlated with viral load. During the same period diminished inflammatory responses and ADP were observed.

The percentage of AMs stayed consistent over the course of SIV infection (Figure 1B). Viral infections can lead to activation of macrophages and subsequent release of factors that recruit additional cells of the innate and adaptive immune system (55, 56). The lack of detection of macrophage recruitment to the lung may have resulted from a concurrent lymphocyte recruitment, making the relative number of macrophages appear consistent. Indeed, a higher percentage of lymphocytes in BAL of HIV-infected patients has been reported (57). Here, estimating actual AM numbers by multiplying cell count with percentage of AMs did not result in significant differences (Figure S2). Including absolute counting beads as part of the initial analysis might have provided more definitive answers. Variations in surface marker expression of AMs over 20 weeks of SIV infection were also not seen, except for changes in FcγRIII expression (Figures 1, 5). Phenotypic comparisons of AMs from the BAL of HIV-infected and uninfected individuals also have not shown differences (57).

Proinflammatory cytokines IL-6 and IL-1β, TNF-α, and chemokine RANTES was induced in AMs during acute infection (Figures 2A–E). The rapid secretion of cytokines and chemokines was expected as the innate immune system initiates lymphocyte recruitment to establish adaptive immunity prior to viral spread. Although the HIV/SIV infection rate of AMs may be low, macrophages respond to exposure to viral particles or virus-derived gene products such as Nef, Tat, and the gp120 envelope (18, 58). Following the acute phase, the elevated cytokine and chemokine responses by AMs were not sustained despite exposure to viral products and likely LPS from the gut (59). Further, stimulation with LPS or native gp120 *ex vivo* showed an impaired IL-6 and MIP-1β response of AMs from chronically infected macaques (Figure 3), indicating a potentially compromised inflammatory response against lung pathogens. This novel observation is consistent with data showing inhibition of TNF-α release by macrophages in response to Toll-like receptor (TLR)-4 stimulation during HIV infection, even though the expression level of TLR-4 remained unchanged (60). Desensitization of TLR ligands on AMs as a result of viral respiratory infections has also been reported (61). The β-Chemokines MIP-1α, MIP-1β, and RANTES can bind to the HIV-1 co-receptor CCR5 indicating a potential protective effect against entry of R5-tropic viruses (62–65). The pattern of chemokine expression here shows that any protective effect of β-chemokines expressed by AMs is likely limited to the acute phase of infection (Figures 2, 3).

The lowering of cytokine and chemokine responses after the acute phase of infection was not associated with increased IL-10 expression (Figure 2F). In fact, AMs of naïve macaques expressed the highest level of intracellular IL-10 (Figure 2F). Some studies have shown no induction of IL-10 secretion or mRNA in macrophage-tropic HIV-1 infected MDM compared to uninfected cells (66). Others have shown increased IL-10 secretion and mRNA levels for *in vitro* HIV-infected PBMC, monocytes and MDMs [reviewed in (67)]. Differences in virus tropism or target cells could account for the variations observed. Increased levels of IL-10 have been detected in the BAL-F of HIV infected patients (68), however the IL-10 in these BAL samples could have originated from other lymphocytes that upregulate IL-10 during HIV infection (69). In sum, our data indicate that SIV disease progression is not associated with increased AM IL-10 expression, suggesting that AMs are not switching to a more regulatory phenotype.

AMs are essential for lung microbial clearance. Despite reports that AM function is not affected by HIV-1 infection (27, 28), recent studies have shown that phagocytic activity can be impaired (26, 70). As viral load and activation status of macrophages can vary over the course of infection, here we tracked the phagocytic activity of AMs following SIV infection, providing new insights into the dynamics of AM function. AMs readily internalized antigen coated beads during acute infection, but this ability markedly decreased in chronically infected animals (Figure 4B). The initial phagocytic activity was non-specific, as no gp120-specific antibody was present, and was likely due to the rise in proinflammatory responses during acute infection (Figures 2A–E). ADP was observed in the chronic stage of infection when gp120-specific antibody levels increased in BAL-F (Figures 4C,D). However, a continued rise in gp120 antibody levels was not associated with increased ADP. In fact, decreased ADP activity was seen later in chronic infection. Therefore, even though the pattern of chemokine expressing AMs over the course of SIV infection (Figures 2A–E) suggested that frequencies returned to naive levels during the chronic phase of infection, the diminished functional activity observed suggested AM dysfunction.

FcγRIII expression was elevated during acute SIV infection but decreased as infection progressed (Figures 5A,D). These data are partly in contrast to data from HIV-infected MDMs that showed either elevated or no change in FcγR expression [reviewed in (6)]. The FcγRIII isoform expressed in macrophages (FcγRIIIa) consists of a ligand bindingα-chain associated with disulfide-linked γ-chains (71, 72). The γ signaling subunit of FcγRs has been shown to be downregulated as a consequence of HIV infection, resulting in aberrant downstream signaling required for phagocytosis (46, 73). The FcγRIII antibody used here recognizes the ligand binding FcγRIII α-chain (74); thus the loss of FcγRIII expression cannot be explained by downregulation of the γ-chain alone. Our data suggest that in spite of an initial increase in the frequency of FcγRIII^+^ AMs during acute infection (Figure 5A), prolonged HIV infection may lead to diminished expression of FcγRIII. This outcome may not have been observed previously as *in vitro* MDM infection experiments mainly mimic acute stages of infection. Nevertheless, although the frequency of FcγRIII^+^ AMs at 12-wpi had dwindled to <30% of the level observed in naïve animals, a correlative trend between the frequency of FcγRIII^+^ AMs and ADP was observed (Figure 5G) indicating the importance of this receptor in ADP activity mediated by AMs. Furthermore, FcγRIII MFI negatively correlated with acute viremia suggesting an important role for FcγRIII-mediated phagocytosis in initial viral load control (Figure 5H).

The PD-1/PD-L pathway plays a key role in negative regulation of adaptive immunity in HIV and other viral infections [reviewed in (75)], but few studies have explored the role of PD-1 in innate immunity, particularly by macrophages. PD-1 expression on T cells following immune activation and its role in T-cell exhaustion when highly expressed during HIV-1 and other chronic viral infections have been described (52, 76). Regarding macrophages, PD-1 expression has been linked to diminished ability to clear microbial invasion in septic mice (77), inhibition of phagocytic activity and tumor immunity (13), and inability to perform phagocytosis and intracellular killing in patients with tuberculosis (78). Unlike on T cells, PD-1 expression on macrophages in these studies described a single positive population. Here, we also identified a single PD-1^+^ population of AMs derived from chronically infected macaques (Figures 6C,D). The macaques were clear of lung infections at the time of BAL collection, suggesting PD-1 expression was only associated with SIV infection. Until now, no direct evidence has implicated PD-1 in AM dysfunction. Our data show a direct correlation between PD-1 and SIV viremia and suggest that in keeping with correlations described with T cells (53), disease progression can also be associated with PD-1 expression in AMs (Figure 6E).

While PD-1 ligands were found to be expressed on AMs from naïve macaques, PD-L1 expression levels were low and subsequently declined after SIV infection (Figure 7A). This result was unexpected and in contrast to a study on MDMs that showed up-regulation of PD-L1 and PD-L2 after exposure to inactivated HIV virions (79). However, the alveolar environment is indeed unique and it is unsurprising that AM responses to SIV infection differ from *in vitro* exposure of MDMs to virions. Rodriguez-Garcia et al. further indicated differential regulation of PD-L1 and PD-L2 by IL-10, whereby presence of IL-10 increased PD-L1 expression and its blockade increased PD-L2 expression (79). Analysis of IL-10 expression by AMs in our study showed a gradual decrease after SIV infection and may be one explanation as to why we observed decreased PD-L1 expression (Figure 2C). PD-L2 expression, however, remained high and did not increase with decreased IL-10 expression. Future analysis of the factors found in BAL-F could provide further insight into the dynamics of PD-L expression on AMs.

PD-1 blockade experiments have shown enhancement of SIV/HIV-specific responses, proliferative ability and cytokine production by exhausted PD-1 high T cells (80–82). In keeping with this, blockade of the PD-1/PD-L pathway has been reported to ameliorate phagocytic function in macrophages found in the tumor microenvironment and in active tuberculosis (78, 83). Here, we also found that blockade of PD-1 could significantly improve phagocytic activity further highlighting PD-1 as a factor playing a role in dysfunction of AMs (Figure 7C). In addition, although not significant potentially due to small sample size, we found lower abundance of TNF-α expressing PD-1^+^ AMs compared to the PD-1^−^ population during chronic infection (Figure 7D). These data suggested that the presence of PD-1 on AMs is likely a factor in any inhibitory role exerted on AMs through the PD-1/PD-L pathway.

In summary, our longitudinal investigation has provided important new information about the consequences of SIV infection on AMs, and in novel findings, propose a role for PD-1, a well-recognized inhibitor of adaptive immune responses, on innate immunity against SIV infection.

## Data Availability

All datasets generated for this study are included in the manuscript and/or the Supplementary Files.

## Ethics Statement

Forty-nine female Indian rhesus macaques used in this study were housed in the NCI Animal Facility, Bethesda, MD, under protocol VB-020. The NCI Facility is accredited by the Association for Assessment and Accreditation of Laboratory Animal Care International, and its Animal Care and Use Committee approved all animal experiments prior to study initiation. Standard practices followed recommendations made in the Guide for the Care and Use of Laboratory Animals of the United States, National Institutes of Health, and the Weatherall report.

## Author Contributions

RH and MR-G: conceptualization. RH, GE-A, and MR-G: methodology. RH and ZM: investigation. RH, ZM, and TH: resources. RH: writing—original draft. RH, ZM, GE-A, TH, and MR-G: writing—review and editing. MR-G: supervision.

### Conflict of Interest Statement

The authors declare that the research was conducted in the absence of any commercial or financial relationships that could be construed as a potential conflict of interest.
