# Supplementary material for: Alveolar Macrophage Dysfunction and Increased PD-1 Expression During Chronic SIV Infection of Rhesus Macaques
Source: Front Immunol. 2019 Jul 3;10:1537. doi: 10.3389/fimmu.2019.01537 (PMC6618664; doi:10.3389/fimmu.2019.01537)
Supplement: Supplementary file 1 [file Data_Sheet_1.PDF]

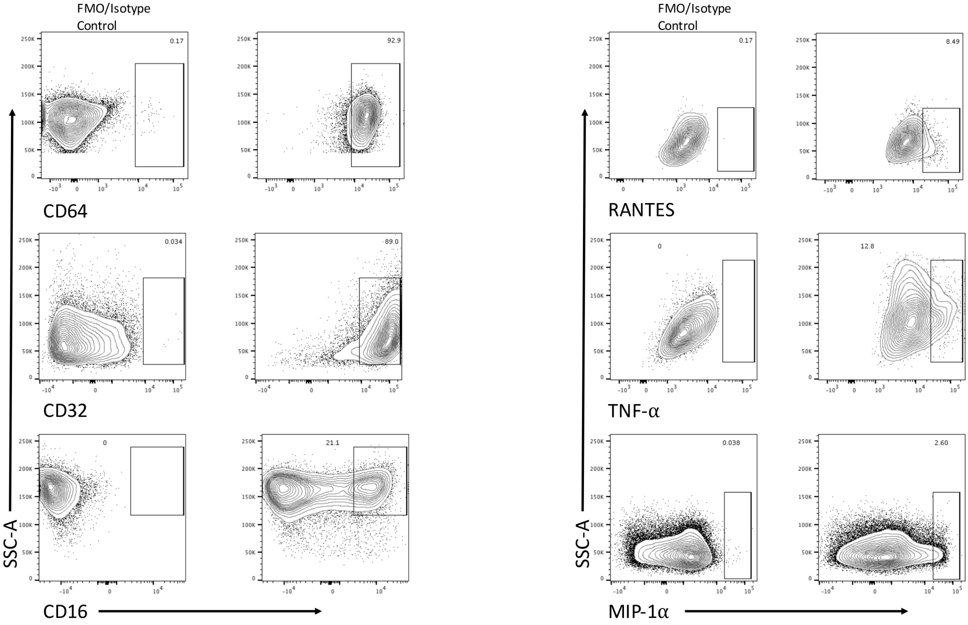

FMO/Isotype Control
CD64
0.17
92.9
CD32
0.034
89.0
CD16
0
21.1
SSC-A
FMO/Isotype Control
RANTES
0.17
8.49
TNF-α
0
12.8
MIP-1α
0.038
2.60
SSC-A

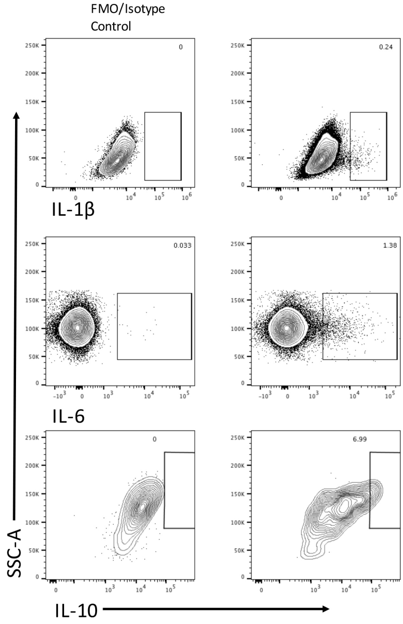

Figure S1. FMO and Isotype controls used to identify positive AM populations

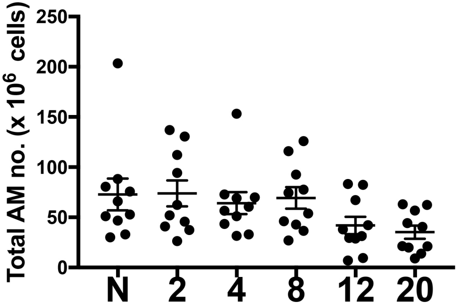

Figure S2. AM count over the course of SIV infection. AM count was calculated as AM frequency (% of leukocytes) X cell count. n = 10
